# Female Urethral Stricture Caused by Lichen Sclerosus: An Uncommon Presentation

**DOI:** 10.7759/cureus.42551

**Published:** 2023-07-27

**Authors:** Deepak Kumar, Ankur Mittal, Vikas K Panwar, Omang Agrawal

**Affiliations:** 1 Urology, All India Institute of Medical Sciences Rishikesh, Rishikesh, IND

**Keywords:** female lichen sclerosus, success rate of urethroplasty, voiding urinary symptoms, female buccal mucosal graft urethroplasty, female urethral stricture disease

## Abstract

Lichen sclerosus is an inflammatory disease of the mucocutaneous region. The etiology of lichen sclerosus is not well explained. Lichen sclerosus is more common in females and generally involves the genital area. Urethral involvement in lichen sclerosus is uncommon in females. Males have a higher propensity for urethral involvement in lichen sclerosus. Here we report a case of a 50-year-old female with lichen sclerosus and urethral stricture. Buccal mucosal graft urethroplasty was done. The success rate of urethroplasty is low in patients with lichen sclerosus. Meatal sparing urethroplasty is usually not recommended in females with urethral strictures associated with lichen sclerosus. Our patient was asymptomatic at six months of follow-up.

## Introduction

Lichen sclerosus (LS) is an inflammatory disease of the mucocutaneous region [1]. It involves the anogenital region in 85%-98% of cases. Extragenital involvement has been seen in 15%-20% of cases [2]. Hallopeau initially reported LS in 1887 under the name "lichen plan atrophique," and Darier later used the phrase "lichen plan scléreux" to describe it histologically. Lichen sclerosus is six to ten times more common in females than in males [3]. Lichen sclerosus can be seen at any age in females, but its prevalence has seen two peaks: the first at prepubertal age and the second at postmenopausal age [4]. The mean age of diagnosis is the fifth decade [1]. The lichen sclerosus of the female genitalia is mainly associated with pruritus, dyspareunia, dysuria, and pain during defecation. 

On examination, white, thinned-out, dry skin with plaque, stenosed introitus, and sometimes fissures and ulcerations of the skin can be seen. Urethral involvement is common in male patients but rare in female patients [5]. Here we are reporting a case of female urethral stricture disease caused by lichen sclerosus.

## Case presentation

A 50-year-old female patient presented to our OPD with a complaint of difficulty in micturition and dyspareunia for the last one year. Symptoms were insidious in onset and gradually progressive. The patient had no history of fever, burning micturition, flank pain, or prolapse. On examination, there was white, dry, atrophic skin present in the genital region involving the labia majora, labia minora, clitoris, and urethral orifice. Uroflowmetry was done and showed a compressive obstructed flow curve (Qmax 3.4ml/sec, Voided volume 225 ml, and PVR 5 ml).

**Figure 1:**
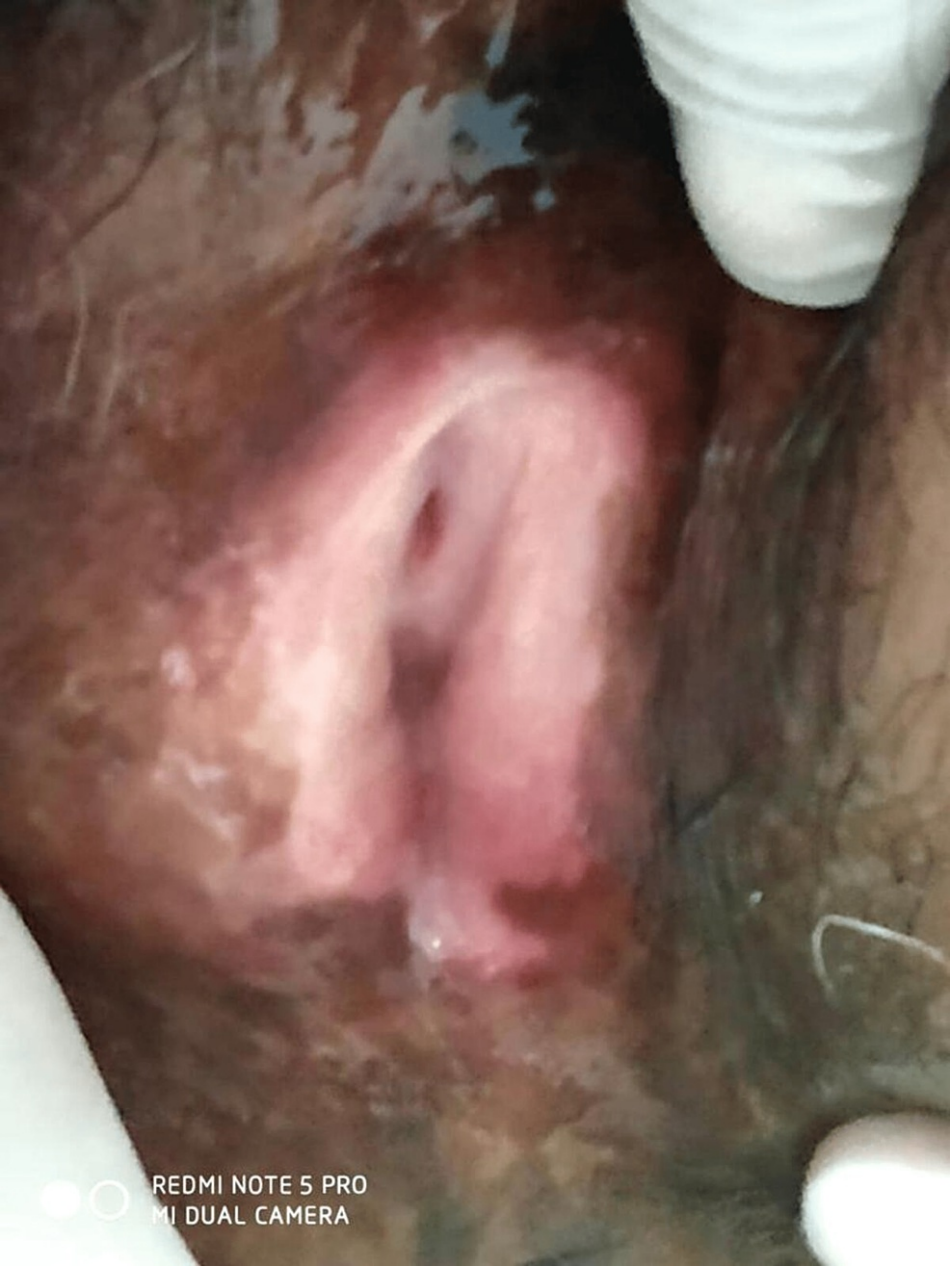
Lichen sclerosis involving the vagina, labia majora, and urethra

All blood investigations were normal. Urethroscopy was done; only a 9F ureteroscope was negotiable in the urethra, and there was a white-scarred stricture seen in the distal 2.5 cm urethra. 

Ultrasonography of the abdomen showed normal bilateral kidneys and urinary bladders.

A biopsy was taken from the vulvar region. The patient was diagnosed with lichen sclerosus with urethral stricture. The topical steroid was started and continued for one month. Dyspareunia was improved, but there was no change in lower urinary tract symptoms. The patient was taken for surgery for the management of urethral structure disease. A lithotomy position was made. The incision was made in the supramental region. The dorsal surface of the urethra was dissected from the surrounding structure. The urethra was incised dorsally until the normal urethra. A buccal mucosal graft was taken and anastomosed dorsally with the urethra. 18 F per urethral catheter was placed and removed after 10 days. 

**Figure 2:**
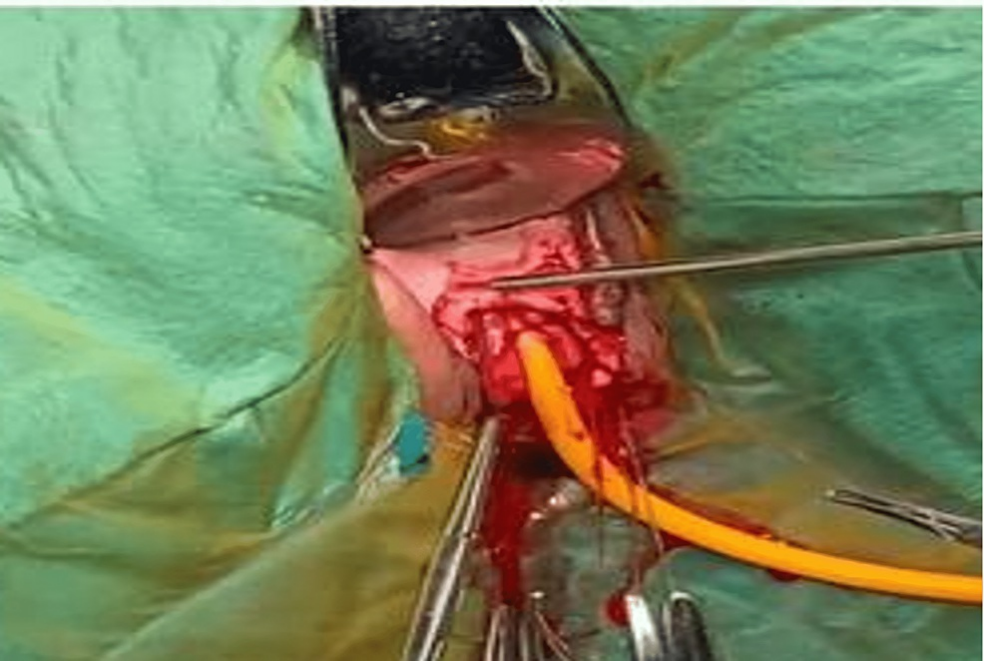
Image showing dorsal onlay buccal mucosal graft urethroplasty

**Figure 3:**
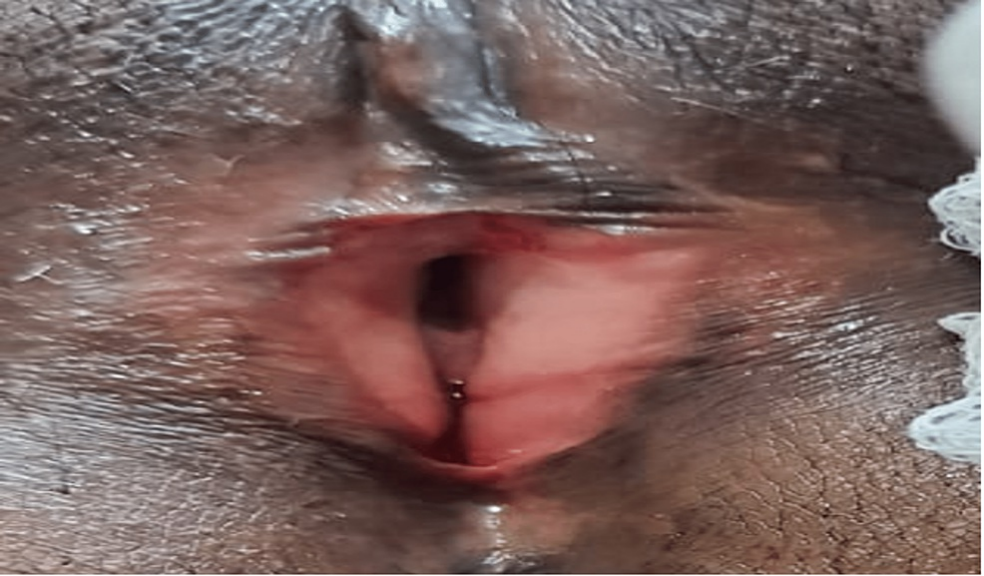
Follow up image taken three months after surgery. A wide open urethral meatus was present.

The patient voided normally after catheter removal. The external urethral meatus was normal in diameter, as shown in figure. 

## Discussion

Lichen sclerosus is also known as lichen sclerosus et atrophicus, lichen albus, and hypoplastic dystrophy [6]. Inflammation and increased fibroblast function lead to fibrosis of the dermis. Cell-mediated immunity also plays a significant role in lichen sclerosus [7].

The etiology of lichen sclerosus is not well explained. LS has been found to be more prevalent among twins and family members. Alopecia areata, vitiligo, thyroid disease, diabetes mellitus type 1, and pernicious anemia are the most common disorders associated with lichen sclerosus. The presence of autoantibodies is common in LS, like extracellular matrix 1 and anti-basement membrane antibodies [8,9]. The diagnosis of lichen sclerosus is clinical, but a biopsy is recommended in suspicious cases. Although lichen sclerosus is not a premalignant condition, patients with lichen sclerosus have a high risk of developing squamous cell carcinoma [10].

20%-30% of women with lichen sclerosus remain asymptomatic [11]. Most patients present with complaints of pruritus and soreness, which aggravate at night. The involvement of the female urethra is an uncommon presentation of lichen sclerosus. The length of the urethral stricture is greater when lichen sclerosus is present. Local tissues are usually not suitable for use in urethroplasty since they are unhealthy. 

Females with urethral involvement in lichen sclerosus typically have urethral meatus involvement; hence, meatal-preserving urethroplasty is typically not advised in cases of urethral stricture brought on by lichen sclerosus in females. Urethral strictures caused by lichen sclerosus have a low success rate. Treatment options for lichen sclerosus include topical steroids, topical calcineurin inhibitors, systemic therapies (including steroids, cyclosporin, and methotrexate), and surgery. Indications for surgery include premalignant lesions, urinary dysfunction, and acute infections. In our case, we started topical therapy. Urinary symptoms didn’t respond to topical therapy. So the patient was planned for Buccal mucosal graft anastomotic urethroplasty.

## Conclusions

Lichen sclerosus is more common in females. The most common presentation in females is pruritus. Although lichen sclerosus is a clinical diagnosis, a biopsy should be considered in suspicious cases.

Urethral involvement by lichen sclerosus in females is an uncommon presentation. Urethral involvement usually does not respond to dilatation or medical therapy. Surgery is the definitive treatment for urethral strictures caused by lichen sclerosus.
